# The HERV-K accessory protein Np9 controls viability and migration of teratocarcinoma cells

**DOI:** 10.1371/journal.pone.0212970

**Published:** 2019-02-28

**Authors:** Susana M. Chan, Tamar Sapir, Sung-Soo Park, Jean-François Rual, Rafael Contreras-Galindo, Orly Reiner, David M. Markovitz

**Affiliations:** 1 Cellular and Molecular Biology Program, University of Michigan, Ann Arbor, Michigan, United States of America; 2 Department of Molecular Genetics, Weizmann Institute of Science, Rehovot, Israel; 3 Department of Pathology, University of Michigan, Ann Arbor, Michigan, United States of America; 4 Division of Infectious Diseases, Department of Internal Medicine, University of Michigan, Ann Arbor, Michigan, United States of America; 5 Graduate Program in Immunology, University of Michigan, Ann Arbor, Michigan, United States of America; 6 Cancer Biology Program, University of Michigan, Ann Arbor, Michigan, United States of America; Hirosaki University Graduate School of Medicine, JAPAN

## Abstract

Human endogenous retroviruses are remnants of ancient germline infections that make up approximately 8% of the modern human genome. The HERV-K (HML-2) family is one of the most recent entrants into the human germline, these viruses appear to be transcriptionally active, and HERV-K viral like particles (VLPs) are found in cell lines from a number of human malignancies. HERV-K VLPs were first found to be produced in teratocarcinoma cell lines, and since then teratocarcinoma has been thought of as the classical model for HERV-Ks, with the NCCIT teratocarcinoma cell line particularly known to produce VLPs. Treatment for teratocarcinoma has progressed since its discovery, with improved prognosis for patients. Since the introduction of platinum based therapy, first year survival has greatly improved even with disseminated disease; however, it is estimated that 20% to 30% of patients present with metastatic germ cell tumor relapse following initial treatments. Also, the toxicity associated with the use of chemotherapeutic agents used to treat germ cell tumors is still a major concern. In this study, we show that the depletion of the HERV-K accessory protein Np9 increases the sensitivity of NCCIT teratocarcinoma cells to bleomycin and cisplatin. While decreasing the expression of Np9 had only a modest effect on the baseline viability of the cells, the reduced expression of Np9 increased the sensitivity of the teratocarcinoma cells to environmental (serum starvation) and chemical (chemotherapeutic) stresses. Np9 is also essential to the migration of NCCIT teratocarcinoma cells: in a wound closure assay, reduced expression of Np9 resulted in cells migrating into the wound at a slower rate, whereas reintroduction of Np9 resulted in NCCIT cells migrating back into the wound in a manner similar to the control. These findings support the implication that the HERV-K accessory protein Np9 has oncogenic potential.

## Introduction

Human endogenous retroviruses (HERVs) account for 8% of the human genome, yet their potential roles in the biology of the cell and in human health or disease remain poorly understood. These ancient viruses were once exogenous viruses that infected germ cells of mammals and other vertebrates numerous times in the course of millions of years, and subsequently integrated their proviral elements into the host genome. These proviruses have then been transmitted over the generations in a Mendelian fashion [1–3]. HERV elements exist in the human genome as retroviral genes (*gag*, *pol*, and *env*) flanked by two long terminal repeats (LTRs); the LTRs serve as transcriptional promoters [4]. However, most of the HERV proviral sequences have been rendered nonfunctional due to the accumulation of mutations, insertions, and deletions in crucial retroviral genes [5], with the possible exception of the HERV-K (HML-2) subfamily. The HERV-K (HML-2) subfamily is one of the most recent entrant into the human genome, most having only integrated itself between 200,000 and 5 million years ago, and it is the subfamily of endogenous retroviruses that is most conserved and are transcriptionally active, and some have functional open reading frames (ORFs) to code for all of its viral proteins [4,6–10].

There are approximately 117 full-length copies and 944 to 1200 solitary LTRs of HERV-K (HML-2) spanning multiple chromosomes [1,11–16]. In addition, our group has discovered that there are at least hundreds of copies of two HERV-K (HML-2) viruses found throughout the centromeres of multiple chromosomes, proviruses that we have termed K111 and K222; both are type-I viruses [15,17]. There are two types of HERV-K (HML-2) viruses: type I is characterized by a 292-bp deletion at the boundary of the *pol* and *env* genes, while type II contains the full sequence for *pol* and *env*. In the type I virus, the 292-bp deletion causes an alternative splicing event that results in the expression of the accessory protein Np9, while type II expresses the accessory protein Rec [4].

The transcription and translation of HERV-K genes and proteins are thought to be tightly repressed under normal physiological conditions, but there is evidence of HERV-K reactivation in different malignancies. For example, even though no infectious HERV particles have been detected to date, HERV-K (HML-2) have been found to produce viral like particles (VLP) in the tumor tissues of patients with breast cancer, leukemia, lymphoma, melanoma, and teratocarcinoma, and in the blood of HIV-infected individuals [10,14,15,18–26]. In addition, the increased transcription and expression of the HERV-K accessory proteins Np9 and Rec are thought to have potentially important roles in cellular functions that may contribute to oncogenesis [27–31].

HERV-K Rec is a 14 kDa protein that is a functional homolog of HIV Rev [32,33]. Rec aids in the shuttling of unspliced mRNA out of the nucleus, and has been associated with tumor development similar to testicular carcinoma in nude mice [29,32]. The HERV-K accessory protein Np9 is a 9 kDa protein that shares its first 14 amino acids with HERV-K Rec, both of which are translated from the HERV-K *env* reading frame [34]. A recent study showed that the *np9* and *rec* transcripts are not restricted to diseased states [35]. However, the actual HERV-K Rec and Np9 accessory proteins appear to be expressed mainly in malignant tissues. Rec and Np9 proteins have been detected in primary and metastatic melanoma biopsies and melanoma cell lines but not found in melanocytes [22,36]. Also, *np9* transcripts have been found in transformed cell lines and tumors such as mammary carcinomas, germ cell tumors, and leukemia blood lymphocytes [34].

The role that these accessory proteins play in promoting oncogenesis is still not well defined. However, there has been some progress in identifying potential interacting partners and the functions of these accessory proteins in different cellular pathways. HERV-K Np9 and Rec have both been shown to physically and functionally interact with the promyelocytic zinc finger (PLZF) tumor suppressor and inhibit its function as a transcriptional repressor. The PLZF tumor suppressor is a known transcriptional repressor of the c-*myc* proto-oncogene. The co-expression of Np9 or Rec with PLZF removes the transcriptional repression of the c-*myc* promoter by PLZF, resulting in the overexpression of c-Myc and altered expression of c-Myc regulated genes, thus effecting cell proliferation and survival [28]. HERV-K Np9 has also been shown to interact with the RING-type E3 ubiquitin ligase LNX (ligand of Numb protein X) [31], and Np9 has been found to play a critical role in different cell signaling pathways by activating β-catenin, ERK, Akt and Notch1 [30]. The expression of Np9 is crucial for the survival and growth of myeloid and lymphoblastic leukemia cells: reduced expression of Np9 caused growth inhibition of myeloid and lymphoblastic leukemia cells, whereas overexpression of Np9 promoted the growth of leukemia cells [30]. Lastly, NOD-SCID mice developed larger tumors at a faster rate when injected subcutaneously with lymphoma cells overexpressing Np9 as compared to mice that received lymphoma cells with a control vector [30]. Further studies are necessary to examine the role of Np9 in other types of tumors.

In the studies presented here, we investigated the function of Np9 in teratocarcinoma, a classical model for HERV-K and cancer. It was in teratocarcinoma cell lines that investigators first saw the production of VLPs, first termed human teratocarcinoma-derived viruses (HTDV), and it was later determined that HERV-K was responsible for encoding HTDV [19,37–39]. HERV-K (HML-2) mRNA and proteins are also highly expressed in teratocarcinoma [34,40]. The aim of the present study was to investigate whether the expression of Np9 supports or promotes tumorigenesis. We show that decreasing expression of Np9 with CRISPR/Cas9 decreases the viability of the NCCIT teratocarcinoma cell line when it is subjected to environmental stress (serum starvation) or chemotherapeutic agents (bleomycin and cisplatin) that are used in clinical settings as a part of a cocktail to treat testicular germ cell tumors (TGCT) [41–46]. Further, the reduced expression of Np9 decreased cell migration and invasiveness of teratocarcinoma cells, and re-introduction of Np9 rescued the migration of the NCCIT teratocarcinoma cells. Thus, we show that Np9 is crucial to the viability and mobility of teratocarcinoma cells, and decreasing its expression can potentiate the effectiveness of chemotherapeutic agents used in the clinic.

## Materials and methods

### Cell lines and cell culture

The teratocarcinoma cell line NCCIT was obtained from ATCC and cultured in RPMI [Thermo Fisher Scientific (TFS), catalog #11875–093], supplemented with 10% fetal bovine serum (FBS) and 1% penicillin-streptomycin (P-S), at 37°C in a 5% CO_2_ cell culture incubator. The medium was changed every 2 to 3 days. Cells were cultured in T-75 Falcon Tissue Culture Treated Flasks (Fisher Scientific, catalog #353112) until 75% confluent and split with TrypLE Express (TFS, catalog #12604013) for 5 min in the 37°C incubator. Cells were re-suspended in RPMI supplemented with 10%FBS and 1% P-S. Cells were counted with Countess Automated Cell Counter (TFS) using Countess Cell Counting Chamber Slides (TFS, catalog #C10228).

### CRISPR/Cas9 plasmid construct and transfection

The CRISPR/Cas9 guide RNAs were designed using the online CRISPR Design Tool (http://crispr.mit.edu) to specifically target HERV-K Np9. The Np9 guide RNAs (gNp9-1: gaacgggccatgatgacga; gNp9-2: ggttttgtcgaaaagaaaag) were cloned into the pSpCas9(BB)-2A-Puro (pX459) V2.0 plasmid. The pSpCas9(BB)-2A-Puro (pX459) V2.0 was a gift from Feng Zhang (Addgene, plasmid #62988). As a control, a scramble sgRNA (gScr: gagatcgagtgccgcatcac) cloned into the pX459 plasmid was used; the scramble plasmid construct was kindly provided by Dr. Xiaoyan Jia.

NCCIT cells were seeded at a density of 1,000,000 cells per well in a CytoOne 6-well tissue culture plate (USA Scientific, catalog # CC7682-7506) and grown for 24 hours. For transfection of pX459 constructs, we incubated FuGENE HD transfection reagent (Promega, catalog #E2311) with OPTI-MEM reduced serum medium (TFS, catalog #31985088), and 500 μg of each plasmid was incorporated at a 1:6 ratio (DNA:Transfection reagent) for 30 min at room temperature before being introduced into cells. After a 24 hour transfection with sgRNAs, the medium was changed to RPMI supplemented with 2 μg/mL puromycin (Sigma, catalog #P8833) for selection, and the NCCIT cells were selected for 3 days and then cultured in RPMI for 2 days to recover. After 2 days of recovery, the NCCIT clones were transfected with two additional rounds of CRISPR/Cas9 constructs. As there are hundreds of copies of Np9 scattered around the genome, some with slightly different sequences, CRISPR/Cas9-mediated genome editing results in knockdown (KD), rather than a knockout cell line.

### Immunoblot analysis

NCCIT cells were treated with 0.3 μM epoxomicin (Millipore, catalog #324800) for 24 hours to allow for the stabilization of Np9. NCCIT cells were collected with a cell scraper and lysed in cold RIPA Buffer (Sigma, catalog #R0278) supplemented with cOMPLETE protease inhibitor cocktail (Roche, catalog # 11697498001). Protein lysates (33 μg protein per sample) were separated in Bio-Rad 4–20% Mini-PROTEAN TGX gels (Bio-Rad, catalog #4561094). Proteins were transferred to 0.45 μm pore size polyvinylidene difluoride membranes (PVDF) (Millipore, catalog # IPVH00010), incubated with anti-Np9 rat monoclonal antibody 10B1 or 22E4 (1:25) overnight at 4°C, and then incubated with rabbit anti-rat secondary antibody (1:5000) (Invitrogen, catalog #619520) [47]. The anti-Np9 rat monoclonal antibodies were kindly provided by Dr. Klaus Römer from the University of Saarland Medical Center. Membranes were incubated with SuperSignal femto chemiluminescent substrate (TFS, catalog #34095). As a loading control, the PVDF membrane was incubated with mouse anti-GAPDH antibody (1:10000) (Santa Cruz, catalog #166545), and then incubated with horse anti-mouse secondary antibody (1:10000) (CST, catalog #7076S). We used ImageJ to analyze the intensity of the Np9 signal to determine the efficiency of the CRISPR/Cas9-mediated gene KD.

### Starvation and MTT assay

To synchronize and starve the teratocarcinoma cells, the NCCIT cells were seeded at a density of 1000 cells per well in a Falcon 96-well tissue culture plate (Fisher, catalog #353072) and cultured in 100 μL RPMI supplemented with 1% P-S for 24 hours. After 24 hours, the medium was removed and changed to 100 μL full RPMI medium (time 0). At time 0, each well was treated with 10 μL MTT (3-(4,5-Dimethylthiazol-2-yl)-2,5-Diphenytltetrazolium Bromide) (5 mg/mL) for 3 hours, and then 100 μL of solubilization solution (10% SDS in 0.01M HCl) was added to each well to stop the reaction. The plate was then wrapped in aluminum foil and incubated in a 37°C/5% CO_2_ incubator overnight, and was analyzed the following day with a Tecan GENios plate reader. Days 4, 5 and 6 were done in similar manner and optical density (OD) measurements were obtained. The optical density measurements were used to calculate percent cell viability, days 4, 5 and 6 were divided by OD obtained on day 0, resulting number was multiplied by 100 to determine percentage viability.

### Bleomycin and cisplatin treatments and MTT assay

NCCIT cells were seeded at a density of 1000 cells per well in Falcon 96-well tissue culture plates (Fisher, catalog #353072) and cultured in full RPMI media for 24 hours. At 24 hours, the media was changed to media supplemented with 5, 15, 30, 60 or 120 μg/mL bleomycin (Cayman Chemical, catalog #13877) or 0.1, 0.5, 1, 15, 15 μg/mL cisplatin (Cayman Chemical, catalog #13119) and the plates were cultured for 48 hours. MTT assay was performed at 0 and 48 hours post-treatment. Calculations were done in a similar manner as previously described.

### Cell cycle analysis

NCCIT cells were seeded at a density of 220,000 cells per well in a 6-well CytoOne tissue culture plate (USA Scientific, catalog #CC7682-7506), cultured in RPMI, and the media was changed every 24 hours. The cells were trypsonized with TrypLE Express, collected and centrifuged at 700 rpm for 5 mins. The cell pellet was washed once with 1X phosphate buffered saline (PBS) (TFS, catalog #10010023) and fixed with 70% ice-cold ethanol for 30 mins on ice. The fixed cells were washed with 1X PBS, re-suspended with FxCycle PI/RNase staining solution (Molecular Probes, catalog #F10797), and stained in the dark for 30 mins prior to analysis. The cells were analyzed with a Bio-Rad ZE5 cell analyzer at the University of Michigan Flow Cytometry Core.

### Flow cytometry for activated caspase 3/7 apoptosis assay

NCCIT cells were seeded at 150,000 cells per well in a 6-well CytoOne tissue culture plate (USA Scientific, catalog #CC7682-7506) and cultured in full RPMI medium for 24 hours. After 24 hours, the medium was changed to mock treatment (full RPMI media) or RPMI medium containing 30 μg/mL bleomycin. After 24 hours, the cells were trypsonized with TrypLE Express, collected, washed with 1X PBS (PBS), and stained with CellEvent Caspase 3/7 Green Detection Reagent (TFS, catalog #C10427) for 60 mins; for the last 5 mins the cells were stained with SYTOX AADvanced Dead Cell Stain (TFS, catalog #C10427). Caspase 3/7 analysis was performed with a Sony SH800 Cell Sorter at the University of Michigan Flow Cytometry Core.

### Wound closure assay

Wound closure assay was performed with NCCIT Np9 CRISPR cells or with control NCCIT scramble cells. The tissue culture plates were treated with 0.001% poly-L-lysine solution (Sigma, catalog # P8920) and stored at 4°C until use. NCCIT cells were seeded at a density of 200,000 cells per well in a 12-well CytoOne tissue culture plate (USA Scientific, catalog #CC77682-7512) and cultured in RPMI for 24 hours. After 24 hours, each well was scratched linearly with a pipette tip, and images were captured at 0 and 24 post-scratch. To determine percent migration, images were measured used to measure the width of the wound through the entire length of the wound within the image using the NIS Elements image software. The percent migration was calculated by dividing the width of the wound at time 24 with time 0 and then multiplying by 100 to obtain percentage of migration.

### Np9 complementation plasmid construct

Re-introduction of Np9 into NCCIT Np9 KD cells was performed using the piggyBac transposon system. Np9 complementation was performed with the PB-CAG-Np9-mCherry transposon, and the PB-CAG-H2B-mCherry transposon was used as the control. NCCIT cells (scramble, Np9 KD #8 and #9) were seeded at a density of 1,000,000 cells per well in a CytoOne 6-well tissue culture plate. NCCIT scramble control cells and NCCIT Np9 KD cell clones 8 and 9 were co-transfected with PB-CAG-H2B-mCherry transposon plasmid and pCAG-PBase transposase plasmid at a 1:1 ratio as a control. NCCIT Np9 KD clones 8 and 9 were co-transfected with PB-CAG-Np9-mCherry transposon and pCAG-PBase transposase plasmid at a 1:1 ratio. Twenty-four hours post transfection, the cells were selected for mCherry using a Sony SH800 cell sorter, and allowed to grow for a week in full media. The cells were selected a second time for mCherry a week after the first selection to ensure that the mCherry expressing constructs were stably integrated. These cells were used for a wound closure assay done in a similar manner as previously described.

## Results

### Np9 controls cellular viability when teratocarcinoma cells are stressed

In view of the potential link between Np9 and teratocarcinoma, we performed loss-of-function studies to investigate the role of Np9 in the viability of the NCCIT teratocarcinoma cell line. We used two independent CRISPR/Cas9 constructs to make permanent cell lines in which Np9 expression had been knocked down. The CRISPR/Cas9 system is an efficient gene editing system that uses guide RNAs specifically designed to target and edit the gene of interest [48]; in our study, we used two independent Np9 guide RNAs to knock-down and reduce the number of Np9 copies in NCCIT teratocarcinoma cells, and isolated individual clones. Typically, the CRISPR/Cas9 system is used to mutate or knock-out (KO) a gene of interest [48,49]. Given the high number of Np9 gene copies in the human genome and the sequence heterogeneity from one Np9 locus to another, the use of CRISPR/Cas9 is expected to result in a partial knock-down. For example, the type I virus K111 discovered by our group can be found in hundreds of copies across 15 different centromeres [14,15,17]. Although the K111 proviruses do not seem to be replication competent, they code for many Np9 proteins, including some variants.

**Fig 1:**
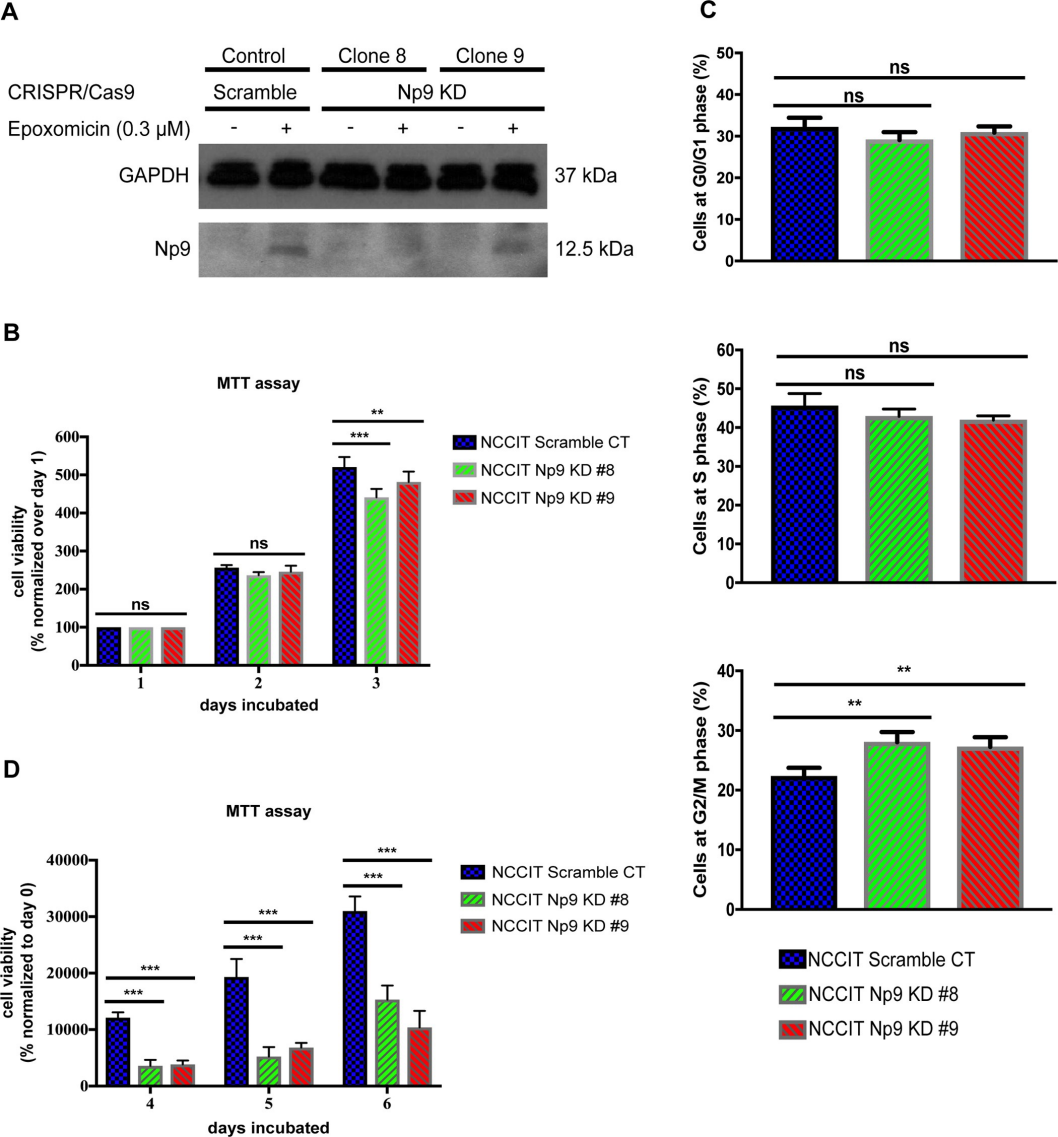
Reduced expression of Np9 in NCCIT teratocarcinoma cells increases sensitivity to environmental stress. (A) NCCIT cells were either transfected with a scramble CRISPR/Cas9 system or Np9 CRISPR/Cas9 guide RNAs. After three rounds of transfection with CRISPR/Cas9 Np9 guides, Clones 8 and 9 were selected, and the level of Np9 was shown to be reduced by 80% and 20%, respectively; Np9 densitometry was analyzed with ImageJ. (B) NCCIT scramble control and Np9 KD cells were cultured in full media and viability was measured with MTT assay (n = 6 for each cell line). Reduced expression of Np9 had a limited effect on the viability of the teratocarcinoma cells. (C) NCCIT scramble control and Np9 KD cells (n = 4 for each cell line) were cultured in full media for 48 hours, and media was changed every 24 hours. Cell cycle analysis was performed with the Bio-Rad ZE5 cell analyzer. Reduced expression of Np9 had a very modest effect on the distribution of the cell cycle in teratocarcinoma cells. (D) NCCIT scramble control and Np9 KD cells were cultured in serum free media (environmental stress) for 24 h, and after 24 h media was changed to full media, and viability was measured with MTT assay (n = 8 for each cell line). The reduced expression of Np9 increased sensitivity to environmental stress and resulted in fewer viable cells. T bars denote the standard deviations of the means; P values were determined using Two-way ANOVA. ns not significant, *P<0.05, **P<0.001, ***P<0.0001.

In our study, we used two independent Np9 guide RNAs to knock-down and reduce the number of Np9 copies in NCCIT teratocarcinoma cells. As can be seen in Fig 1A, knocking down the expression of Np9 was successful. However, the reduced expression of Np9 varied among the different clones that were isolated. In the two knock-down clones presented in this study, the expression of Np9 was reduced approximately by 80% and 20% in the knock-down clones 8 and 9, respectively. Np9 protein expression can only be seen after treatment with epoxomicin to inhibit the proteasome pathway, as Np9 typically has a very short half-life [31]. Even though Np9 is a 9 kDa protein, it has been shown to be represented as a 12.5 kDa signal [47], perhaps due to post-translational modifications. When Np9 was knocked down, we observed that it had a modest effect on the growth of NCCIT cells as represented by the MTT assay, which measures metabolic activity (Fig 1B). When we further examined the effect of Np9 knockdown in this teratocarcinoma cell line, consistent with the modest impact on cell viability we found only a small alteration in the cell cycle distribution, more so in Np9 KD clone 8 that causes a greater reduction in Np9 expression (Fig 1C). However, many oncoproteins, as well as other biologically crucial molecules, exert their effects primarily under duress. Therefore, we subjected the knock-down and control cells to serum starvation, and found a marked reduction in cellular metabolism in the two knock-down clones (KD clone 8 and clone 9) as compared to a clone in which only a scramble CRISPR/Cas9 guide RNA was used (Fig 1D). Therefore, taken together, we see that the reduction in the levels of Np9 does have a significant effect on the growth and metabolism of this type of cancer cell, but primarily when the cells are put under stress. This prompted us to move on to examine the effect of Np9 knock-down in the setting of chemotherapeutic agents used in the clinic.

### Decreased Np9 expression increases sensitivity of teratocarcinoma cells to chemotherapeutic agents

**Fig 2:**
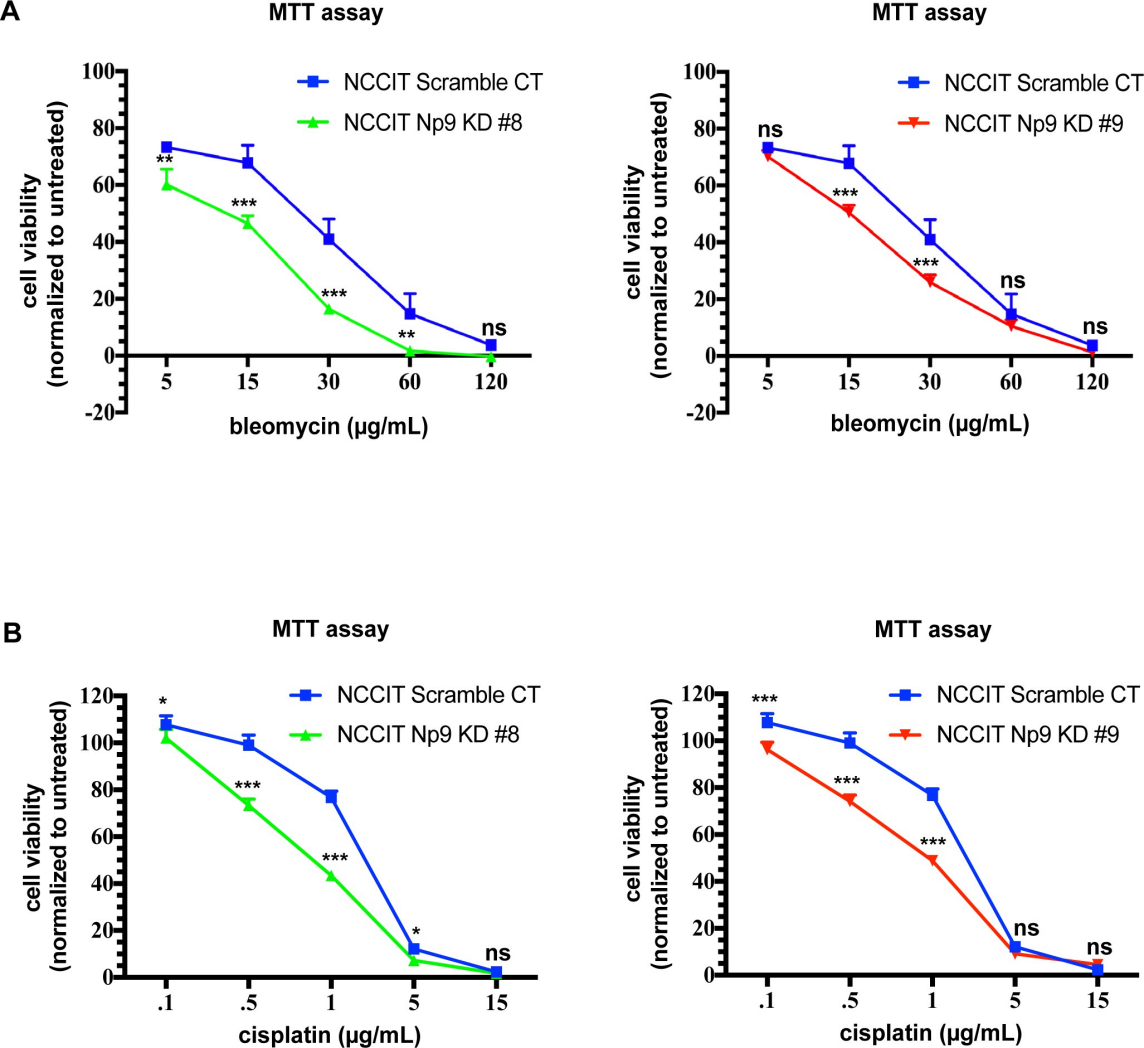
Reduced expression of Np9 in NCCIT teratocarcinoma cells increases sensitivity to chemical stress. NCCIT scramble control and Np9 KD cells were cultured in regular media for 24 h (n = 4 for each cell line). The following day, the media was changed to mock treatment (full media) or media supplemented with (A) bleomycin (30 μg/mL) or (B) cisplatin (1 μg/mL) and cultured for 48 h; at 48 h viability was measured with MTT assay. Reduced expression of Np9 resulted in increased sensitivity to bleomycin or cisplatin treatment with decreased viability of NCCIT cells. T bars denote the standard deviations of the means; P values were determined using student t-test analysis. ns not significant, *P<0.05, **P<0.001, ***P<0.0001.

In the clinical setting, testicular teratocarcinomas are frequently treated with combinations that include bleomycin and cisplatin, two highly toxic agents [41–43,45,46]. As decreasing Np9 expression sensitized NCCIT cells to starvation stress, we next investigated whether cells that had Np9 expression knocked down would be more susceptible to chemical stress induced by bleomycin and cisplatin. Indeed, when Np9 expression was diminished NCCIT cells treated with bleomycin (Fig 2A) or cisplatin (Fig 2B) for 48 hours showed markedly reduced cell viability. This loss of viability was seen with both Np9 KD clones, as compared to the scramble control.

**Fig 3:**
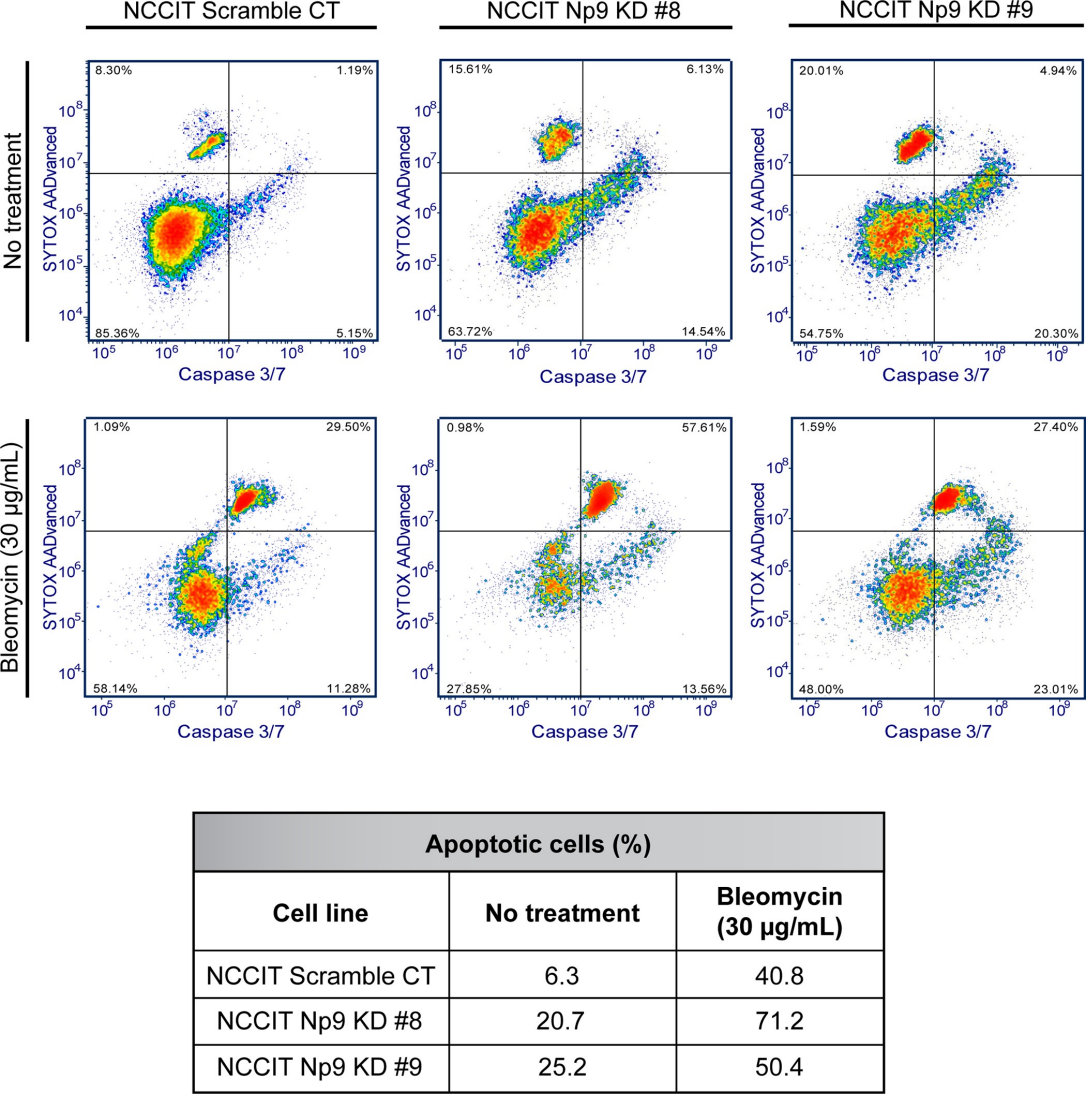
Reduced expression of Np9 in NCCIT teratocarcinoma cells increases sensitivity to chemical stress, resulting in apoptotic cells. NCCIT scramble control and Np9 KD cells were cultured in full media. The following day media was changed to either mock treatment (full media) or treatment (30 μg/mL bleomycin). After 24 h of treatment, the cells were stained with SYTOX AADvanced and Caspase 3/7 and apoptosis analysis was performed with a Sony SH800 Cell Sorter. Np9 KD clones 8 and 9 were naturally more apoptotic than scramble control cells: 20 and 25% compared to 6%, respectively. The reduction of Np9 expression in NCCIT cells increased sensitivity to bleomycin, with increased apoptosis in Np9 KD clones 8 and 9 compared to scramble control cells: 71% and 50% compared to 40%, respectively. The percent of apoptotic cells represented in the table was calculated from the bottom right and upper right quadrants, with the bottom right quadrant being early stage apoptotic cells and the upper right quadrant being late stage apoptotic cells.

By appearance, the NCCIT cells in which Np9 was knocked-down appeared to be apoptotic through the loss of adhesion. We therefore stained NCCIT cells (KDs and Scr control) with activated Caspase 3/7 green detection dye and SYTOX AADvanced viability dye to discriminate early and late stage apoptotic cells from live and dead cells. We found that, in spite of the limited effect on overall growth and metabolism as measured by MTT assay (Fig 1B), diminished Np9 expression did lead to some increase in apoptosis even in the absence of any stress (Fig 3). However, this increase in apoptosis was magnified when bleomycin was applied to the cells (Fig 3).

### Decreasing Np9 levels severely affects migration of NCCIT cells

**Fig 4:**
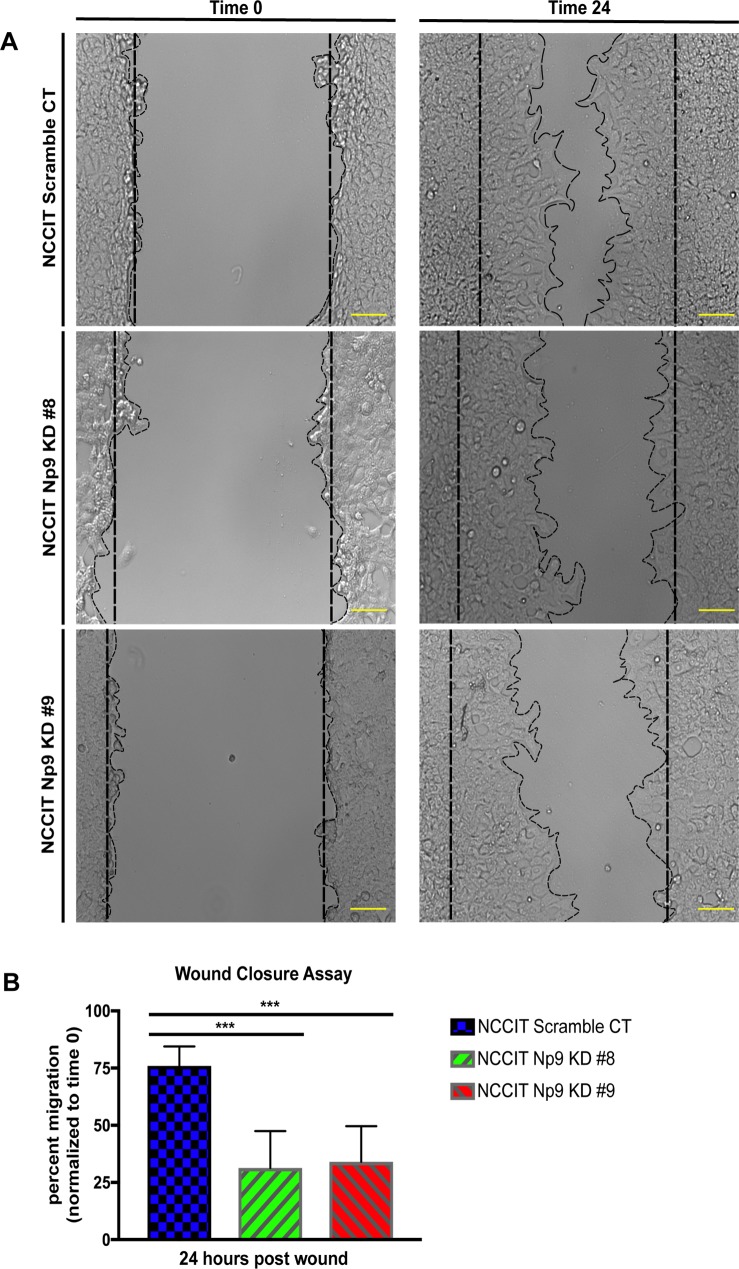
Np9 is essential for cell migration and wound closure. (A) NCCIT scramble control and NCCIT Np9 KD clones 8 and 9 were used for the migration assay. A total of 200,000 cells for each cell type were transferred into a 12-well plate. The following day, a scratch was created down the middle, and pictures were taken at time 0 and at 24 hours. The images are representative of 3 independent experiments done in duplicate. Scales bars, 100 μm. (B) The wound was measured at time 0 and at 24 hours. The percent of migration was plotted over time 0. The measurements are representative of 3 independent experiments done in duplicate. The width of the wounds was measured in each image and totaled for scramble CT (n = 26), KD #8 (n = 27), and KD #9 (n = 28). The reduction in the expression of Np9 in NCCIT cells resulted in reduced cell migration post-wound as compared to scramble control cells. T bars denote the standard deviations of the means; P values were determined using student t-test analysis. ***P<0.0001.

The data above demonstrate that the effect of Np9 on the growth, metabolism, and viability of teratocarcinoma cells is particularly visible when the cells are put under stress. We therefore wanted to examine another aspect of oncogenesis that reflects cell migration, and so employed a wound-closure assay. In this assay, a scratch is induced to cells growing in a monolayer and then the time it takes for the “wound” to close is monitored. This is one important test for examining the contribution of a cellular protein to the oncogenic process [50,51]. As can be seen in images in Fig 4A and graphically in 4B, knocking down Np9 expression with CRISPR/Cas9 led to a marked slowing in wound closure. At 24 hours (Fig 4B), the wound closure was only approximately 25% to 50%, as opposed to somewhere between 75% and 90% in the control cells. Therefore, a decrease in Np9 expression clearly affects the ability of cells to migrate. It is important to note that it is difficult to tease apart migration from cell viability. However, the wound closure assay is generally considered to influence migration more particularly than viability.

**Fig 5:**
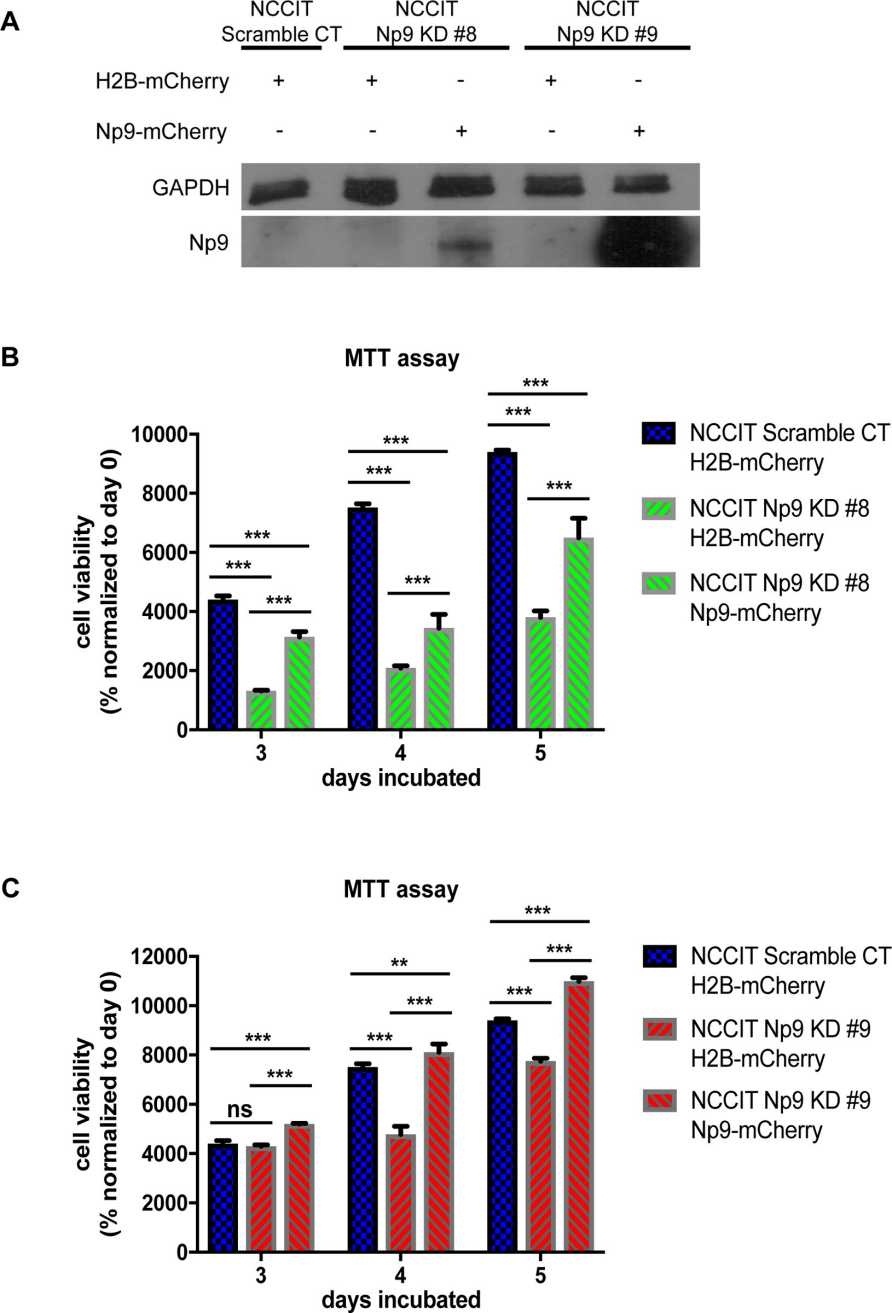
Re-introduction of Np9 into teratocarcinoma cells rescues viability in the presence of environmental stress. (A) Re-introduction of Np9 was performed using the piggybac system. NCCIT scramble CT, Np9 KD #8 and Np9 KD #9 cells were either transfected with a PB-CAG-H2B-mCherry control transposon or PB-CAG-Np9-mCherry transposon, and a pCAG-PBase transposase. The cells were selected twice for mCherry expression using the Sony SH800 cell sorter. To visualize Np9 re-introduction, NCCIT cells were treated with 0.3 μM of Epoxomicin for 24 hours and 33 μg of protein per lane was analyzed by western blotting, using rat monoclonal anti-Np9 antibody 22E4 (1:25) and mouse monoclonal anti-GAPDH antibody (1:10,000). (B) and (C) NCCIT scramble H2B-mCherry, Np9 KD#8 and #9 H2B-mCherry, and Np9 KD #8 and #9 Np9-mCherry cells were cultured in serum free media (environmental stress) for 24 h, and after 24 h media was changed to full media, and viability was measured with MTT assay (n = 4 for each cell line sample size; results shown are representative of two separate experiments). The re-introduction of Np9 rescued the viability of the cells in serum starved conditions. T bars denote the standard deviations of the means; P values were determined using Two-way ANOVA. ns = not significant, *P<0.05, **P<0.001, ***P<0.0001.

**Fig 6:**
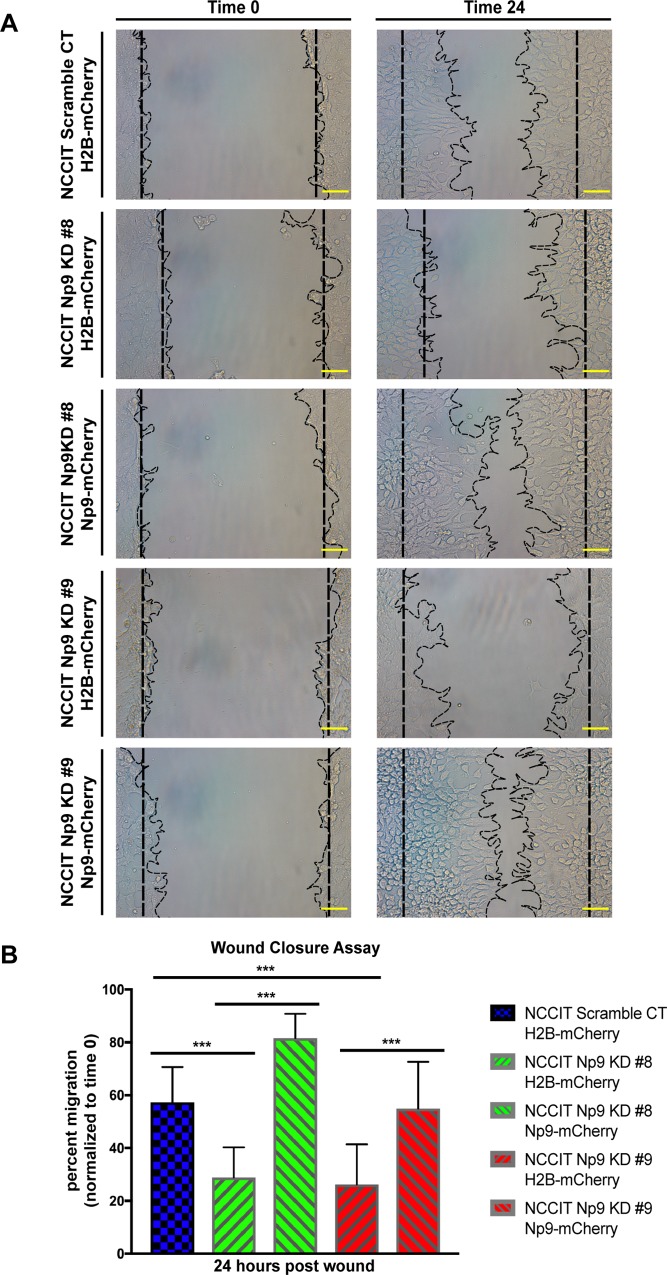
Re-introduction of Np9 into teratocarcinoma cells rescues the migration phenotype. (A) NCCIT scramble, Np9 KD clones 8 and 9 with H2B-mCherry control clones and NCCIT Np9 KD clones 8 and 9 with Np9-mCherry clones were used for the migration assay. A total of 200,000 cells for each cell type were transferred into a 12-well plate. The following day, a scratch was created down the middle, and pictures were taken at time 0 and at 24 hours. The images are representative of 2 independent experiments done in duplicate. Scales bars, 100 μm. (B) The wound was measured at time 0 and at 24 hours. The percent of migration was plotted over time 0. The measurements are representative of 2 independent experiments done in duplicate. The width of the wound was measured for each sample: scramble CT-H2B-mCherry (n = 83), Np9 KD #8 H2B-mCherry (n = 135), Np9 KD #8 Np9-mCherry (n = 156), Np9 KD #9 H2B-mCherry (n = 160), and Np9 KD #9 Np9-mCherry (n = 145). The re-introduction of Np9 into Np9 KD cells rescued the migratory phenotype of the NCCIT cells almost to the level of the scramble control, and in the case of Np9 KD clone 8 complemented with Np9-mCherry, the re-introduction of Np9 resulted in faster migration compared to the scramble control. T bars denote the standard deviations of the means; P values were determined using student t-test analysis. ***P<0.0001.

In order to confirm that Np9 is crucial to cell viability and wound healing, we employed the piggybac system to stably overexpress the Np9-mCherry fusion protein; the mCherry was used as a visual indicator for the stable expression of Np9. Western blot analysis was performed to visualize the re-introduction of Np9 (Fig 5A). The re-introduction of Np9 into NCCIT Np9 KD clones 8 and 9 resulted in the rescue of viability when cells were subjected to environmental stress (serum starvation) (Fig 5B and 5C). Strikingly, when we overexpress Np9 in the NCCIT cells in which Np9 expression has been reduced with CRISPR/Cas9 editing, the wound healing is restored essentially to normal. This is seen in the pictures in Fig 6A and is depicted graphically in Fig 6B, showing that we were able to restore migratory function of the teratocarcinoma cells by reintroducing Np9. This rescue experiment offers striking evidence that Np9 is important to wound closure in NCCIT cells, and further suggests an important role for this protein in the growth and movement that is necessary for oncogenesis.

## Discussion

HERVs have long been implicated in autoimmune disorders and oncogenesis [10,20,24,36,52–66]. However, the fact that there are so many copies, over a hundred copies of the standard HERVs and hundreds to thousands of copies of K111 and K222, picking apart the specific contributions of HERV-K to cancer has been quite difficult [15,17]. Adding to the complexity of HERV-K biology in the context of human diseases, there is evidence of HERV-K viral particle production in certain cancers and transformed cell lines, such as teratocarcinoma; however, whether these viral particles are actually replicative remains unknown and the subject of debate [14,16,18–22,67–72].

In the field of HERV-K biology, the Np9 protein has been of particular interest in view of its potential to function as an oncogene. Indeed, Np9 has been shown to control cellular signal transduction pathways that modulate expression of growth genes such as Notch, Akt, Wnt/ß-catenin, and Myc [28,30,31]. Further, it has been shown to interact with the promyelocytic leukemia zinc finger tumor suppressor [28]. In the Notch pathway, Np9 has been shown to interact with Numb, and has been thought to perhaps stimulate Notch signaling via this interaction, although the details have remained quite unclear [31]. Here, we offer strong evidence that reducing the expression of Np9 decreases the cell viability of NCCIT cells, especially when they are challenged by serum starvation or chemotherapeutic agents. The effect is primarily mediated through apoptosis, although the mechanistic effect of combining the cisplatin agent with Np9 knock-down remains unclear. Perhaps most remarkably, we find using wound healing assays that Np9 is vital for the proliferation/invasion phenotype, further strengthening our observations that Np9 is important for the growth and likely oncogenic properties of teratocarcinoma cells.

Teratocarcinoma is often a treatable disease, but the treatment regimens are often quite toxic. Furthermore, the relapse rate is high in advanced stage disease [41,44,46]. Here we show that a reduction in Np9 sensitizes NCCIT cells to the clinically important chemotherapeutic agents bleomycin and cisplatin. Therefore, our data would suggest that targeting Np9 in teratocarcinoma will lead to better outcomes if proper drugs/delivery mechanisms can be obtained. As HERV-K and Np9 have also been implicated in other malignancies [14,15,18,19,21,22,24,36,73], it is certainly possible that targeting Np9 may sensitize these other cancers to chemotherapeutic agents. Taken together, Np9 appears to be a key protein propelling teratocarcinoma viability and a potentially interesting target for future therapeutics.
